# *Lbx2 *regulates formation of myofibrils

**DOI:** 10.1186/1471-213X-9-13

**Published:** 2009-02-12

**Authors:** Haruki Ochi, Monte Westerfield

**Affiliations:** 1 Institute of Neuroscience, University of Oregon, Eugene, OR 97403-1254, USA; 2 Developmental Genomics Research Group, Nara Institute of Science and Technology, 8916-5 Takayama Ikoma Nara 630-0192, Japan

## Abstract

**Background:**

Skeletal muscle differentiation requires assembly of contractile proteins into organized myofibrils. The *Drosophila ladybird homeobox *gene (*lad*) functions in founder cells of the segmental border muscle to promote myoblast fusion and muscle shaping. Tetrapods have two homologous genes (*Lbx*). Lbx1 functions in migration and/or proliferation of hypaxial myoblasts, whereas the function of Lbx2 is poorly understood.

**Results:**

To elucidate the role of Lbx in vertebrate myogenesis, we examined Lbx function in zebrafish. Zebrafish *lbx2 *transcripts appear in newly formed paraxial mesoderm and become restricted to adaxial cells, precursors of slow muscle. Slow muscles lose *lbx2 *expression as they differentiate, while a subset of differentiating fast muscle cells transiently expresses *lbx2*. Fin and hyoid muscle express *lbx2 *later. In contrast, *lbx1b *expression first appears lateral to the somites at late segmentation stages and is later restricted to fin muscle. Morpholino knockdown of Lbx1b and Lbx2 suppresses hypaxial muscle development. Moreover, knockdown of Lbx2 results in malformation of muscle fibers and reduced fusion of fast precursors, although no obvious effects on induction or specification are observed. Expression of myofilament genes, including *actin *and *myosin*, requires the engrailed repressor domain of Lbx2.

**Conclusion:**

Our results elucidate a new function of Lbx2 as a regulator of myofibril formation.

## Background

Skeletal muscle progenitors are specified as a population of multipotent mesodermal cells. These cells subsequently commit to a muscle fate and differentiate into muscle fibers by cell fusion and assembly of contractile myofibrils composed of myosin thick filaments and actin thin filaments. Although the molecular mechanisms that regulate induction of muscle progenitors, commitment of these cells to the muscle lineage, and myoblast fusion are well characterized [1-5], relatively less is known about the mechanisms that regulate the formation of myofibrils [6,7].

The ladybird (Lbx) protein is a member of the homeobox transcription factor family, characterized by an N-terminal engrailed repressor domain. In *Drosophila*, *lbx *is expressed by progenitor cells, founder cells, and syncytial precursors of a larval somatic muscle, the segmental border muscle (SBM, muscle 8) [8,9]. Embryos lacking *lbx *function have missing muscle fibers, unfused myoblasts, or abnormally shaped cells in the SBM. Ectopic *lbx *expression leads to the formation of enlarged or duplicated SBMs [8]. Recent studies showed that Lbx is also required for establishment of morphological, ultrastructural, and functional properties of leg muscles in *Drosophila *[10]. Thus, the *lbx *gene functions in fusion of myoblasts, development of muscle fibers, and the establishment of muscle morphology in *Drosophila*.

In mouse, two *Lbx *genes, *Lbx1 *and *Lbx2*, have also been identified [11]. *Lbx1 *is expressed in a subset of hypaxial myoblasts that migrate into the limbs, tongue, and diaphragm. Lbx2 is expressed in the central nervous system, in neural crest derived structures such as dorsal root ganglia and other parts of the peripheral nervous system, and in the urogenital system [11]. Loss of Lbx1 function results in lack of specific limb musculature, attributed to migration defects [12,13]. Although, Lbx2 knock out mice develop relatively normally, one study suggested that Lbx2 functions in neural-derived tissues under the regulation of Pax3 [14]. Lbx1 has also been studied in *Xenopus*, where it represses *myod *expression and promotes myoblast proliferation before the onset of terminal differentiation [15]. Thus in both mammals and frogs, Lbx1 is thought to function in migrating myoblasts, whereas functions of Lbx2 are still unclear [14].

Many aspects of the molecular pathway that regulates myogenesis are conserved in *Drosophila *and vertebrates [5,16]. However, because studies of vertebrate Lbx have focused mainly on migration or proliferation of myoblasts in hypaxial myogenesis, little is known about whether Lbx functions in other aspects of muscle development that have been characterized in *Drosophila*, such as fusion of myoblasts, formation of fibers, or the establishment of muscle shapes. To study these potential roles of Lbx in vertebrates, we examined *lbx *gene function in zebrafish. Zebrafish axial skeletal muscles contain four fiber types; slow muscle, muscle pioneers, fast muscle, and medial fast fibers. Slow muscle cells are located superficially just under the skin, with fast muscle cells located deeper [1]. Muscle pioneers are located near the horizontal myoseptum that separates dorsal and ventral parts of the myotomes, and medial fast fibers are also located medially in the somite. We previously showed that adaxial cells located adjacent to the notochord are precursors of slow muscle cells and muscle pioneers [17,18]. Adaxial cells are specified to form slow or muscle pioneers by Hh signaling from the notochord [19], Then, slow muscle precursors migrate to the lateral surface of the somite [18]. After the somite forms, non-adaxial muscle precursors in the segmental plate differentiate into fast muscle cells. During this period, both slow and fast muscle precursors are dynamically rearranged in the somite [20,21].

The zebrafish genome contains three *lbx *genes [22].*lbx2*, previously named *lbx1*, is expressed in the ventral region of the somite, in the hindbrain, and in the fin bud [23]. *lbx1a *is expressed in the nervous system and fin bud [24]. Analysis of *lbx1b *expression in zebrafish has not been previously reported[22].

Here, we show that zebrafish *lbx2 *expression first appears at late gastrula stages (70%–80% epiboly) in cells near the margin that later form head muscle and pronephros. Subsequently, *lbx2 *expression is present in the paraxial mesoderm and adaxial cells, which are the precursors of slow muscle and muscle pioneers [1,18]. Although a subset of adaxial cells migrates into the somite during segmentation stages[18], *lbx2 *is not detected in slow muscle cells during or after their migration. As development proceeds to late segmentation stages, a subset of fast muscle cells in both dorsal and ventral regions of the somite turns on *lbx2 *expression that subsequently disappears by the second day of development. Thus, myoblasts in both the slow and fast muscle lineages express *lbx2 *transiently. As development proceeds to the end of the second day, *lbx2 *expression is detected in fin bud and hyoid, similar to Xenopus *lbx1 *expression. In contrast to *lbx2*, *lbx1b *expression is not detected during gastrulation, but first appears in the hindbrain and caudal regions of the neural tube around the 5-somite stage. As development proceeds, a subset of cells lateral to the somites (presumptive fin bud) begins to express *lbx1b*, whereas somite cells never express *lbx1b*. Later, *lbx1b *expression is present in the fin buds. Thus, myoblasts only in fin lineages express *lbx1b*. Morpholino analysis shows that both Lbx1b and Lbx2 function in hypaxial myogenesis. In addition, although Lbx2 is not required for induction of myogenesis or specification of zebrafish skeletal muscle cell fates, Lbx2 is required for normal fusion of fast muscle precursors, as in *Drosophila*. Lbx2 also plays an important role in the formation of myofibrils by regulating expression of filament genes, such as *myosin *and *actin*. We further demonstrate that the engrailed repressor domain of the Lbx2 protein is required for this induction of filament genes. Thus, although previous studies in vertebrates have suggested that Lbx1 functions in proliferation and migration of muscle precursors, our results demonstrate that vertebrate Lbx2 also plays an important role as a regulator of muscle cell differentiation.

## Results

### Slow and fast muscle precursors transiently express *lbx2 *mRNA

**Figure 1:**
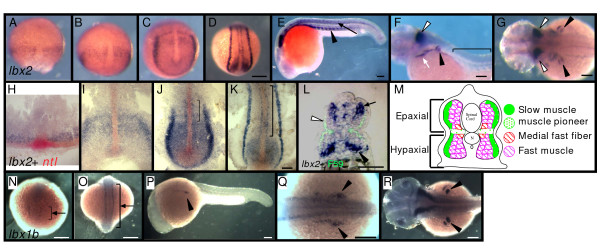
**Muscle precursors express *lbx1b *and *lbx2***. **A-L**: Muscle precursors transiently express *lbx2***. A-G, L**: Expression of *lbx2 *at 70%-epiboly (A), 90%-epiboly (B), bud (C), segmentation (D), 24 hpf (E, L), 48 hfp (F, G). Whole-mounts. **H-K**: *lbx2 *(blue) and *ntl *(red) expression at 70%-epiboly (H), 90%-epiboly (I), bud (J), and segmentation (K). Flat-mounts. **L**: Transverse section, 24 hpf embryo, F59 (green) and *lbx2 *mRNA (blue). (A, H) *lbx2 *mRNA appears at 70%-epiboly adjacent to *ntl *expressing cells in blastoderm margin. *lbx2 *expression in paraxial mesoderm by end of gastrulation (B-C, I,). *lbx2 *expression later restricted to adaxial cells (J-K, brackets). (L) Subset of fast muscle cells expresses *lbx2 *in epaxial (black arrow) and hypaxial (black arrowhead) domains. F59 and *lbx2 *labeling shows differentiated slow muscle cells lose *lbx2 *expression (L, white arrowhead). (F) *lbx2 *expression in trunk disappears by 48 hpf (F, bracket). *lbx2 *expression in fin primordia (F-G, black arrowheads), hindbrain (F-G, white arrowhead), and hyoid (F, white arrow). **M**: Diagram of zebrafish muscle. Adaxial cells (K, bracket) migrate superficially and differentiate into slow muscle fibers (green). Then, fast (magenta) and medial fast fibers (red) differentiate. **N-R**: 5-somite (N), 18-somite (O), 24 hpf (P-Q) and 48 hpf (R). (Q) Higher magnification of P. *lbx1b *mRNA appears at 5-somite stage in neural tube (N), along rostral-caudal axis (O, arrow), then lateral to somites (P-Q), and later in fin (R). (A-D, N-O) Dorsal views, rostral towards the top; (E-F, P) lateral views, rostral toward the left, dorsal toward the top; (H-K) rostral toward the top; (L) dorsal toward the top; (G, R) dorsal views, rostral toward the left. Scale bars: (A-D, N-O, Q) 200 μm, (E-G, P, R) 100 μm, (H-L) 50 μm.

To study the function of *lbx *genes in vertebrate skeletal muscle development, we examined expression of *lbx1b *and *lbx2 *in zebrafish. Previous analysis of *lbx1a *[24] showed that it is not expressed in the somites, so we excluded it from this study. *lbx2 *expression becomes detectable by the 70%–80% epiboly stage (Fig. 1A). Double labeling with *no tail *(*ntl*), which marks the blastoderm margin, reveals that *lbx2 *first appears adjacent to the margin (Fig. 1H) in the region that later contributes to head muscle and pronephros [25]. By the end of bud stage, *lbx2 *expression appears in paraxial mesoderm and adaxial cells (Fig. 1C, J, brackets), precursors of slow muscle cells and muscle pioneers [18]. *lbx2 *expression is not detected in the somites (Fig. 1D, K, rostral region), even though a subset of adaxial cells, the slow muscle precursors, migrates through the somites during segmentation stages [18,20,21].

To learn whether differentiated slow muscle cells express *lbx2*, we double-labeled embryos for *lbx2 *mRNA and with the F59 antibody, a marker of slow muscle [18]. We find that differentiated slow muscle cells do not express *lbx2 *(Fig. 1L, white arrowhead). Instead, *lbx2 *expression appears in the fast muscle domain by late segmentation stages (Fig. 1E, L). Interestingly, we find that both dorsal and ventral fast muscle cells express *lbx2 *(Fig. 1E, L, black arrow and arrowhead respectively), whereas previous studies reported that *lbx2 expression is restricted to ventral regions of the somite *[23]. By 48 hours post-fertilization (hpf), *lbx2 *disappears from the trunk (Fig. 1F, bracket) and appears in fin muscles as previously described (Fig. 1F, G, black arrowheads)[13,23]. *lbx2 *is also expressed in a dorsal intermediate region of the neural tube and hindbrain (Fig. 1F, G, white arrowhead)[15,23] and in the hyoid (Fig. 1F white arrow)[15,23].

Unlike *lbx2*, *lbx1b *expression is not detected during gastrulation. *lbx1b *expression first appears within a dorsal intermediate region of the neural tube by the 5-somite stage (Fig. 1N, arrow), and this expression extends along the rostral caudal axis by late segmentation stages (Fig. 1O, arrow). As development proceeds, a group of cells lateral to the somites (presumptive fin bud) begins to express *lbx1b *(Fig, 1P, Q, arrowhead), whereas muscle precursors in the somites do not express *lbx1b*. Subsequently, *lbx1b *expression is detected in the fin bud (Fig. 1R, arrowheads). Unlike *lbx2*, the hyoid does not express *lbx1b *(Fig. 1R). Thus, during myogenesis, *lbx2 *expression appears in paraxial mesoderm, precursors of slow and fast muscles of the somite, fin bud, and hyoid, whereas *lbx1b *is detected only in myoblast lineages of the fin.

### Conserved function of *lbx *in hypaxial muscle development

**Figure 2:**
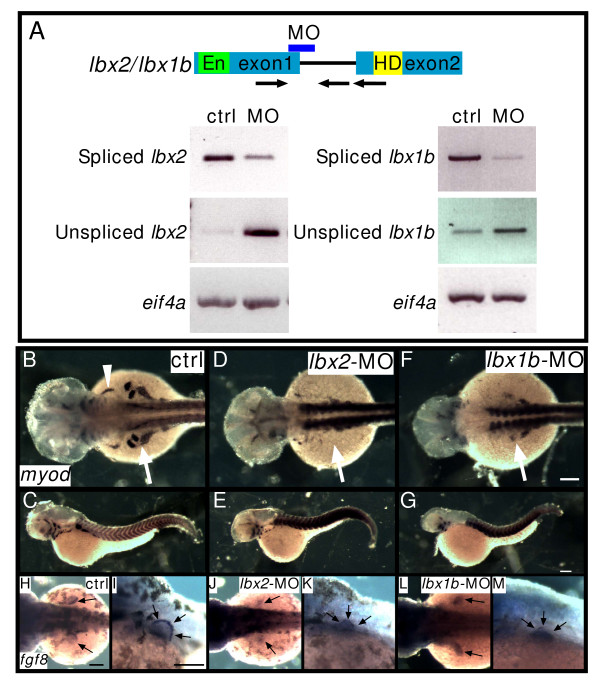
**Lbx2 and Lbx1b function in hypaxial muscle development**. **A**: Splice donor MOs against *lbx2 *or *lbx1b *inhibit correct splicing of *lbx2 *or *lbx1b*, respectively. RT-PCR was performed using bud stage (*lbx2*) or segmentation stage (*lbx1b*) embryos. Spliced bands and unspliced bands in the *lbx-splice donor*-MO lane indicate aberrantly spliced message increases and correctly spliced message decreases. Arrows indicate primers. En: Engrailed domain, HD: homeodomain. Arrows indicate specific primers for amplification of correctly spliced or unspliced *lbx *genes. **B-G**: Expression of *myod *in control (ctrl) embryos (B, C), *lbx2*-MO injected embryos (D, E). *lbx1b*-MO injected embryos (F, G). The white arrowhead indicates the sternohyoideus primordium (B) and the white arrows indicate fin muscle precursors. *myod *expression in fin bud is suppressed by *lbx2*-MO or *lbx1b*-MO (B: 100%, n = 36; D: 12%, n = 56; F: 16%, n = 24). **H-M**: Expression of *fgf8 *in ectodermal cells of the fin bud. Control embryos (H, I, 100%, n = 12), *lbx2*-MO injected embryos (J-K, 100%, n = 12), *lbx1b*-MO injected embryos (L-M, 100%, n = 15). (B, D, F, H, J, L) Dorsal views, rostral towards the left, (C, E, G. I, K, M) lateral views, rostral toward the left, dorsal toward the top. Scale bar: (B-M) 100 μm.

To examine Lbx function in myogenesis, we knocked down Lbx2 or Lbx1b activity using specific splice blocking morpholino oligonucleotides (MOs). To confirm the specificity of the MOs, we performed reverse transcriptase PCR on mRNA from MO injected embryos. We find that injection of *lbx2 *or *lbx1b *splice donor MO results in production of aberrantly spliced *lbx2 *or *lbx1b *transcripts, respectively (Fig. 2A), indicating that splicing of *lbx2 *and *lbx1b *is blocked by the specific *lbx *splice donor MOs.

Previous studies suggested that Lbx1 regulates the migration of limb, hypoglossal, and head muscle precursors in mouse and *Xenopus *[12,13,15]. Our observation that zebrafish *lbx2 *appears in fin bud and hyoid muscles suggests that Lbx2 may be involved in regulation of hypaxial and head muscle development. We find that inactivation of Lbx2 blocks formation of the fin bud and blocks *myod *expression in hyoid muscles (Fig. 2.B–E, arrows and arrowheads respectively). Embryos injected with *lbx2*-MO express *fgf8 *normally in the ectoderm (Fig. 2H–K, arrows)[26], indicating that other aspects of fin bud development are unaffected by MO injection. In addition to *lbx2*, zebrafish *lbx1b *is also expressed in the fin bud (Fig. 1P–R). Injection of *lbx1b*-MO results in decreased *myod *expression in the fin bud, and again, *fgf8 *expression is normal (Fig. 2F, G, L, M). Thus, zebrafish Lbx2 and Lbx1b are both required for proper development of hypaxial muscles that are derived from *lbx2 *and/or *lbx1b *expressing myoblasts, similar to *Xenopus *and mouse *Lbx1 *[12,13,15].

### Functional roles of Lbx2 in the induction of myogenesis and the migration of slow muscle precursors

**Figure 3:**
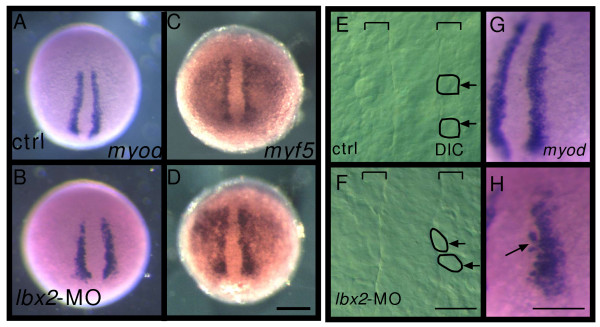
**Lbx2 is not required for the induction of myogenesis**. **A-D**: Suppression of Lbx2 activity does not affect *myod *or *myf5 *expression. The adaxial cells (precursors of slow muscles and muscle pioneers) express *myod *(A, 100%, n = 20). Embryos injected with *lbx2*-MO express *myod *at normal levels (B, 100%, n = 20). The paraxial mesoderm expresses *myf5 *in control embryos (C, 100%, n = 17) and *lbx2*-MO injected embryos (D, 100%, n = 12). **E-F**: Adaxial cell morphology at 3-somite stage in control (E) or *lbx2*-MO injected (F) embryos. Nomarski images of dorsal views. The brackets indicate the width of the adaxial cell row. The normally cuboidal adaxial cells are aberrantly shaped in the *lbx2*-MO injected embryo. Arrows indicate individual adaxial cells. **G-H**: Several *myod *expressing cells fail to incorporate properly into the adaxial cell monolayer after Lbx2 knockdown. (A-H) Whole-mount embryos, dorsal views, rostral toward the top. Scale bar: (A-D) 200 μm (E, F) 25 μm, (G, H) 50 μm.

Our observation of transient expression of *lbx2 *in paraxial mesoderm and adaxial cells at the end of bud stage suggests that Lbx2 might be involved in specification of muscle cells at this stage. [27]. However, we find no obvious changes in expression of myogenic regulatory factors such as *myod *expression in adaxial cells or *myf5 *expression in paraxial mesoderm in *lbx2 *knock down embryos (Fig. 3A–D), although a few *myod *expressing cells fail to incorporate properly into the adaxial cell layer (Fig. 3H, arrow) and the distinctive cuboidal shape of the adaxial cell pseudo-epithelium is somewhat disrupted in (Fig. 3E, F, arrow). Transplantation analysis showed that, although morphogenesis of cells lacking Lbx2 activity is affected, they still express *myod *when located adjacent to the notochord (see Additional file 1). These results indicate that Lbx2 is not involved in the induction of myogenesis.

**Figure 4:**
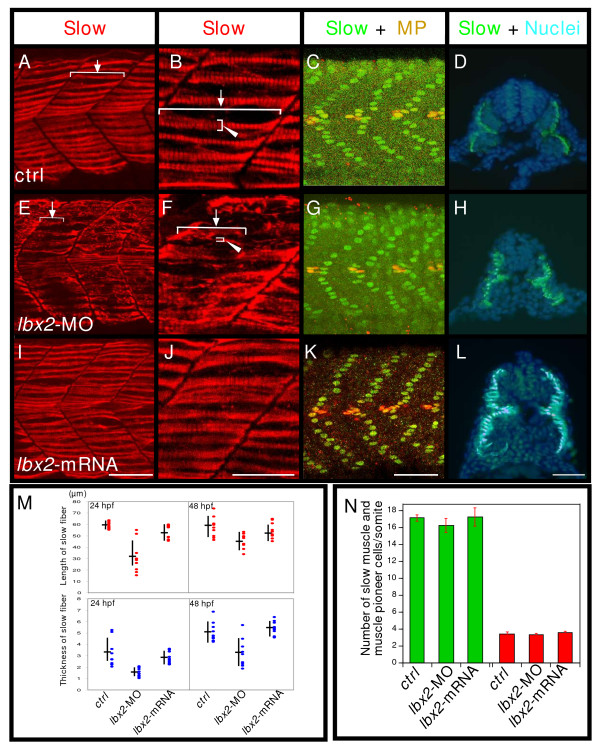
**Knockdown of Lbx2 activity results in malformation of slow muscle fibers**. **A-D**: Control embryos. **E-H**: *lbx2*-MO injected embryos. **I-L**: *lbx2 *mRNA injected embryos. (A-B, E-F, I-J) Embryos labeled with the slow muscle marker, F59 (red). (B, F, J) Higher magnification. Rostral-caudal extension of slow fibers is severely affected by *lbx2 *knockdown (B, F, arrows). **M**: Statistical analyses of mean filament rostral-caudal length (arrows) and dorsoventral thickness (B, F, arrowheads). Analysis by ANOVA demonstrates significant differences in length (P < 0.01) and thickness (P < 0.05). (C, G, K, N) Embryos labeled with Prox1 (green, nuclear slow muscle and muscle pioneer marker), and 4D9 (red, Eng, muscle pioneer marker,). Green indicates slow muscle cells and yellow shows muscle pioneers (MP). (ctrl: n = 10, *lbx2*-MO: n = 6, *lbx2*-mRNA: n = 4). No apparent differences can be detected between controls and embryos injected with *lbx2*-MO. **N**: Analysis by ANOVA demonstrates no significant differences. The data represent the average +/- s.e.m. (D, H, L) Transverse sections of a 24 hpf embryo labeled with the slow muscle marker, F59 (green) and Hoechst to mark nuclei. Slow muscle cells migrate properly to the superficial layer. (A-C, E-G, I-K) Lateral views, rostral toward the left, dorsal toward the top; (D, H, L) dorsal toward the top. Scale bar: (A, C-E, G-I, K-L) 50 μm; (B, F, J) 25 μm.

During segmentation stages, the slow muscle precursors, a subset of adaxial cells, migrate radially away from the notochord to form the superficial layer of slow muscle [18]. Because adaxial cells transiently express *lbx2 *(Fig. 1J, K), we examined whether Lbx2 is involved in their migration. Cross sections of *lbx2*-MO injected embryos indicate that muscle precursors migrate properly through the somite to form the superficial layer (Fig. 4D, H, L). Transplantation analysis also showed that cells lacking Lbx2 function migrate properly to the superficial layer in uninjected embryos (not shown). Thus, in the absence of Lbx2 activity, mesodermal cells form muscle precursors, as indicated by *myod *and *myf5 *expression, and slow muscle precursors migrate normally to the lateral surface of the somite.

### Absence of Lbx2 activity results in malformed slow muscle cells

To explore the functions of Lbx2 further, we examined the number and structure of differentiated slow muscle fibers. Double labeling with Prox1 (labels all slow muscle cell nuclei, [28]) and 4D9 (engrailed, labels muscle pioneer nuclei and medial fast fiber cells, [29]) demonstrated that the number of slow muscle (green) and muscle pioneer cells (yellow) is unaffected by injection of *lbx2 *splice donor MO or *lbx *mRNA (Fig. 4C, G, K, N), indicating normal proliferation of slow muscle precursors and differentiation of slow muscle precursors into slow muscle and muscle pioneers. Thus, the specification and numbers of slow muscle cells and muscle pioneers are regulated independently of Lbx2. On the other hand, however, we find that *lbx2 *splice donor MO injected embryos fail to form normally shaped slow muscle fibers (Fig. 4A, B, E, F, I, J, M). Injection of *lbx2 *translation blocking MO produced the same phenotypes as *lbx2 *splice donor MO (see Additional file 2). Further analysis showed that although slow muscle cells normally contain extended and thickened myofibrils by 24 hpf (Fig. 4B, M and see Additional file 3; length: 60 +/- 0.8 μm, arrow; thickness: 3.52 +/- 0.4 μm, arrowhead; average +/- s.e.m.), *lbx2 *splice donor MO injected embryos exhibit shorter and thinner myofibrils (Fig. 4F, M; length: 32 +/- 4.5 μm, thickness: 1.5 +/- 0.1 μm, Fig. 4M). These malformed slow muscle fibers are still present at 48 hpf (Fig. 4M and see Additional file 4; control: 58 +/- 2.3 μm, 5.1 +/- 0.3 μm; *lbx2 *splice donor MO injected embryos: 46 +/- 1.5 μm, 3.27 +/- 0.4 μm). It is unlikely that these defects are due to a general developmental delay because other aspects of development, including muscle precursor cell migration (Fig. 4H) and rostral caudal extension of the somites (Fig. 4E) occur normally. Thus, Lbx2 contributes to formation of myofibrils in slow muscle cells.

### Fast muscle fibers form abnormal in the absence of Lbx2

**Figure 5:**
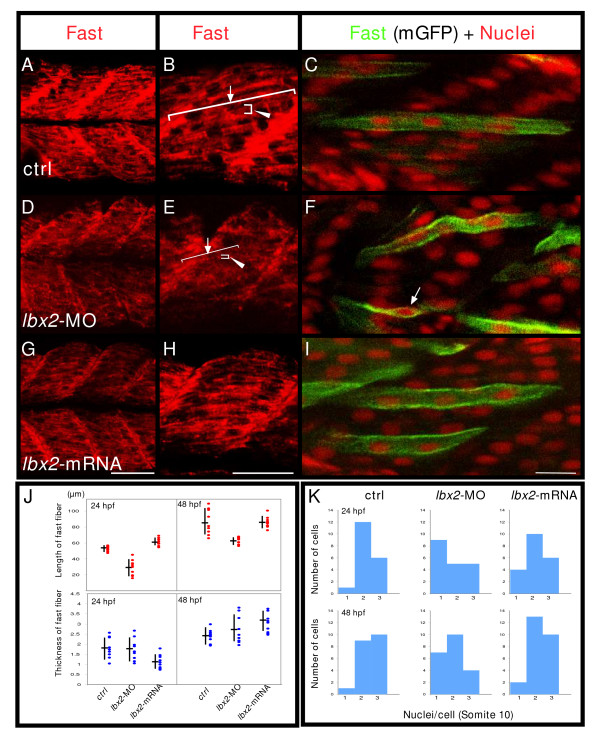
**Fast muscle fibers are malformed in the absence of Lbx2**. **A-C**: Control embryos. **D-F**: *lbx2*-MO injected embryos. **G-I**: *lbx2 *mRNA injected embryos. (A, B, D, E, G, H, J) Embryos labeled with the fast muscle marker, EB165 (red). (B, E, H) Higher magnification views. Rostral caudal extension of fast fibers is severely affected (B, E, arrow). **J**: Statistical analyses of mean filament rostral-caudal length (B, E, arrows) or dorsal ventral thickness (B, E, arrowhead). Analysis by ANOVA demonstrates significant differences in length (P < 0.01). (C, F, I) Embryos were mosaically labeled with membrane localized GFP (mGFP) by injection of DNA and labeled for nuclei (red, propidium iodide). mGFP expressing cells in the fast muscle domain at the level of somite 10 were randomly selected and the number of their nuclei counted. **K**: Fast muscle cells are multinucleate by 48 hpf in both control and *lbx2 *mRNA injected embryos, whereas unfused fast muscle cells are observed in *lbx2*-MO injected embryo (F, arrow). Analysis demonstrates significant differences among the three groups at 48 hpf (Kruskal-Wallis test, p = 0.0121). No apparent differences can be detected between controls and embryos injected with *lbx2*-MO at 24 hpf (Kruskal-Wallis test, p = 0.1509). (A-I) Lateral views, rostral toward the left, dorsal toward the top. Scale bars: (A, B, D, E, G, H) 50 μm, (C, F, I) 20 μm.

Because fast muscle cells transiently express *lbx2 *around 24 hpf (Fig. 1E, L), we also examined the structure of fast muscle cells. The EB165 antibody specifically recognizes fast myosin heavy chain protein (MyHC)[30]. We find that, although fast muscle cells in control embryos contain extended myofibrils by 24 hpf (length: 52 +/- 1.1 μm.), *lbx2 *splice donor MO injected embryos form shorter myofibrils (length: 29 +/- 3.1 μm; Fig. 5A, B, D, E, G, H, J), and these shortened myofibrils are still present at 48 hpf (control: 84 +/- 5.4 μm, *lbx2*-MO injected embryos: 61 +/- 1.9 μm). However, we observe no apparent difference in thickness between control and *lbx2 *splice donor MO injected embryos (24 hpf, control: 1.9 +/- 0.2 μm, *lbx2*-MO injected embryo: 1.8 +/- 0.2 μm; 48 hpf, control: 2.4 +/- 0.1 μm, *lbx2*-MO injected embryo: 2.6 +/- 0.2 μm). Thus, formation of myofibrils in fast muscle cells also depends upon Lbx2 function.

In zebrafish, fast muscle cell precursors are fusing by 24 hpf, and the majority of fast muscle cells are multinucleate [2]. In *Drosophila *that lack Lbx function, myoblasts fail to fuse [8]. Therefore, we examined whether fusion of fast muscle cells is affected by knockdown of Lbx2 activity in zebrafish. Fast muscle cells in somite 10 are multinucleate by 24 hpf and 48 hpf in both control and *lbx2*-mRNA injected embryos, whereas many fast muscle cells remain unfused in *lbx2 *splice donor MO injected embryos (Fig. 5C, F, I, K; the number of nuclei at 48 hpf is significantly different, Kruskal-Wallis test, p = 0.012, see Additional file 5). In addition, unfused cells sometimes exhibit a curved shape. Thus, both the formation of myofibrils and the fusion of fast muscle precursors depend upon Lbx2 activity.

### Interference with Lbx2 activity downregulates myofilament gene expression

**Figure 6:**
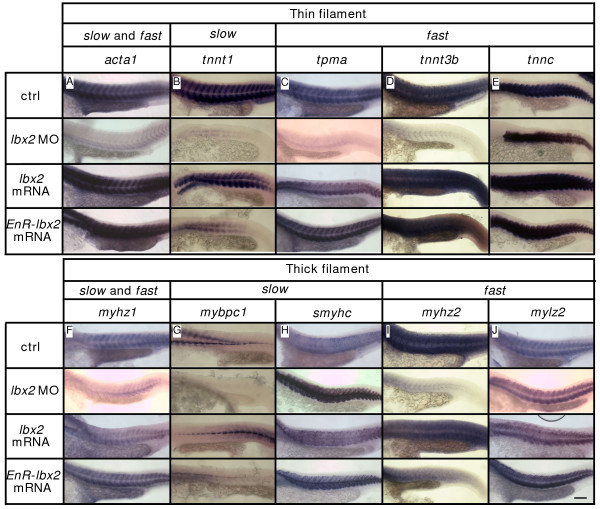
**Expression of thin and thick myofilament genes is downregulated by interference with Lbx2 activity**. **A-J**: Analysis of myofilament gene expression. Expression of *acta1 *(A), *tnnt1 *(B), *tpma *(C), *tnnt3b *(D), *tnnc *(E), *myhz1 *(F), *mybpc1 *(G), *smyhc *(H), *myhz2 *(I), *mylz2 *(J) in control, *lbx2*-MO injected, *lbx2 *mRNA injected, and *Engrailed suppresser domain fused lbx2 *(*EnR-lbx2*) mRNA injected embryos. Expression of *acta1 *(A, ctrl: 28/28, *lbx2*-MO: 0/33, *lbx2*-mRNA: 9/10, *EnR-lbx2 *mRNA: 8/8, numerator indicates number of embryos with normal expression, and denominator indicates number of examined embryos), *tnnt1 *(B, ctrl: 19/19, *lbx2*-MO: 7/22, *lbx2*-mRNA: 12/12, *EnR-lbx2 *mRNA: 17/17), *tpma *(C, ctrl: 16/16, *lbx2*-MO: 2/20, *lbx2*-mRNA: 13/14, *EnR-lbx2 *mRNA: 10/11) and *tnnt3b *(D, ctrl: 13/13, *lbx2*-MO: 0/7, *lbx2*-mRNA: 7/7, *EnR-lbx2 *mRNA: 4/4) is reduced by *lbx2*-MO, but not *tnnc *(E, ctrl: 17/17, *lbx2*-MO: 18/22, *lbx2*-mRNA: 10/10, *EnR-lbx2 *mRNA: 16/16). Expression of *myhz1 *(F, ctrl: 25/25, *lbx2*-MO: 3/16, *lbx2*-mRNA: 7/8, *EnR-lbx2 *mRNA: 8/8), *mybpc1 *(G, ctrl: 15/15, *lbx2*-MO: 10/20, *lbx2*-mRNA: 13/14, *EnR-lbx2 *mRNA: 5/8) and *myhz2 *(I, ctrl: 17/17, *lbx2*-MO: 0/5, *lbx2*-mRNA: 9/9, *EnR-lbx2 *mRNA: 8/8) is reduced by *lbx2*-MO, but *smyhc *(H, ctrl: 36/36, *lbx2*-MO: 24/29, *lbx2*-mRNA: 8/8, *EnR-lbx2 *mRNA: 12/12) and *mylz2 *are unaltered (J, ctrl: 15/15, *lbx2*-MO: 14/15, *lbx2*-mRNA: 10/10, *EnR-lbx2 *mRNA: 7/7). No obvious difference can be detected between embryos injected with *lbx2 *mRNA and embryos injected with *EnR-lbx2 *mRNA. (A-J) Whole mounts, lateral views, rostral toward the left, dorsal toward the top. Scale bar: 100 μm.

Our finding that knockdown of Lbx2 activity results in malformed slow and fast myofibrils suggested that expression of genes encoding components of the sarcomere may depend upon Lbx2 function. We therefore examined the expression of genes encoding components of thin, thick, and elastic filaments. *skeletal muscle alpha-actin *(*acta1*)*, skeletal muscle troponin T *(*tnnt1*)*, fast skeletal muscle tropomyosin *(*tpma*), *skeletal fast troponin T3b *(*tnntt3b*), and *fast skeletal muscle troponin C *(*tnnc*) encode thin filament proteins [24,31]. Both slow and fast muscle cells express acta1, slow muscle cells express tnnt1, and fast muscle cells express tnnt3b [31]. We find that expression of the thin filament genes, *acta1, tnnt1, tpma*, and *tnnt3b *is reduced in Lbx2 deficient skeletal muscles, although *tnnc *expression appears normal (Fig. 6A–E). *skeletal muscle myosin heavy chain *(*myhz1*), slow type myosin binding protein C (mybpc1)*, slow myosin heavy chain 1 *(*smyhc1*), *fast muscle specific myosin heavy polypeptide 2 (myhz2), and fast skeletal muscle myosin light chain 2 *(*mylz2*)*encode *thick filament proteins [7,31,32]. Slow muscle cells, including muscle pioneers, specifically express *mybpc1 *[7], and fast muscle cells express myhz2 [31]. Expression of the thick filament genes, *myhz1, mybpc1*, and *myhz2 *is reduced in embryos lacking Lbx2, whereas *smyhc *and *mylz2 *expression is normal (Fig. 6F–J). titin encodes a component of elastic filaments [33]. In contrast to thin and thick filaments, expression of titin is not affected by lbx2-MO. Thus, deficiency of Lbx2 activity results in decreased expression of specific thin and thick filament genes.

### The engrailed domain of Lbx2 is required for regulation of myofilament gene expression

**Figure 7:**
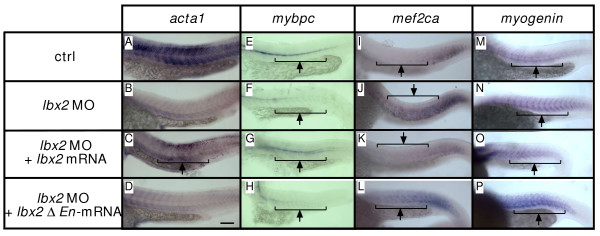
**The Engrailed domain of Lbx2 is required for myofilament gene expression**. **A-H**: Expression of *acta1 *(A-D) and *mybpc1 *(E-H). Embryos express *acta1*at 24 hpf (A: 93% with normal expression, n = 15). Injection of *lbx2*-MO results in decreased *acta1 *expression (B: 20% with normal expression, n = 20), whereas *acta1 *expression partially recovers in embryos injected with *lbx2*-MO + *lbx2 *mRNA (C: 42% with normal expression, n = 50). In contrast, embryos injected with *lbx2*-MO + *lbx *Δ *En *mRNA fail to express *acta1 *(D: 14% with normal expression, n = 37). *mybpc1 *(E: 94%, n = 18; F: 43%, n = 23; G: 81%, n = 31; H: 42%, n = 38). **I-P**: Expression of *mef2ca *(I-L) and *myogenin *(M-P). Embryos express *mef2ca *at 24 hpf (I: n = 11). Injection of *lbx2*-MO results in increased *mef2ca *expression (J: 72% with increased expression, n = 11), whereas *mef2ca *expression partially recovers in embryos injected with *lbx2*-MO + *lbx2 *mRNA (K: 21% with increased expression, n = 19). In contrast, embryos injected with *lbx2*-MO + *lbx *Δ *En *mRNA fail to express *mef2ca *(L: 61% with increased expression, n = 21). *myogenin *(M: n = 7, N: 78% with increased expression, n = 9, O: 23%, n = 13, P: 67%, n = 14). (A-P) Whole mounts, lateral views, rostral toward the left, dorsal toward the top. Scale bar: 100 μm.

In *Xenopus*, overexpression of Lbx1 causes a decrease of *myoD *expression and an increase of *myf5 *expression in hypaxial myoblasts, and the engrailed repressor domain of Lbx1 is required for this function [15]. To learn whether the engrailed domain of zebrafish Lbx2 is required for myofilament formation, we tested whether Lbx2 protein that lacks the engrailed domain, rescues *lbx2*-MO injected embryos. *lbx2*-MO results in decreased *atca1 *and *mybp1 *expression (Fig. 7B, F) and *lbx2 *mRNA rescues *atca1 *and *mybp1 *expression in *lbx2*-MO injected embryos (Fig. 7C, G, arrow). However, mRNA encoding Lbx2 that lacks the engrailed domain fails to rescue *atca1 *and *mybp1 *expression in *lbx2*-MO injected embryos (Fig. 7D, H). In contrast to *atca1 *and *mybp1, mef2c *and *myogenin *expression is upregulated by *lbx2*-MO injection (Fig. 7I, J, M, N) and suppressed by overexpression of *lbx2 *mRNA (see Additional file 6). *lbx2 *mRNA rescues *mef2c *and *myogenin *in *lbx2*-MO injected embryos (Fig. 7K, O, arrow). However, mRNA encoding Lbx2 that lacks the engrailed domain fails to rescue *mef2c *and *myogenin *in *lbx2*-MO injected embryos. Thus, the engrailed domain of Lbx2 is required for Lbx2 function during the formation of myofilaments.

## Discussion

Previous studies suggested that Lbx1 regulates the migration of limb and hypoglossal myoblasts in mouse [12,13]. Recently, an additional function of Lbx1 was reported, whereby Lbx1 represses *myod *expression and promotes myoblast proliferation before the onset of terminal differentiation [15]. In contrast, functions of Lbx2 are poorly understood, because Lbx2 deficiency does not impair mouse development [14]. Here, we demonstrate in zebrafish that *lbx2 *is expressed in mesodermal cells, migratory slow muscle precursors, fin bud, and hyoid [23], whereas *lbx1b *is expressed only in fin bud during myogenesis. Loss of Lbx2 function does not apparently affect induction of myogenesis, slow muscle migration, or proliferation, but rather reduces cell fusion, as in *Drosophila*, and, furthermore, Lbx2 knockdown blocks differentiation of myofibrils due to reduced expression of specific thin and thick filament genes.

### Slow and fast muscle precursor cells transiently express Lbx2

*lbx2 *expression appears by the end of bud stage in paraxial mesoderm and in adaxial cells, the precursors of slow muscle including muscle pioneers. A subset of adaxial cells migrates through the somite during segmentation stages [18], but *lbx2 *is not expressed in nor required for migration of these cells. Interestingly, a subset of fast muscle cells begins to express *lbx2 *in both epaxial and hypaxial domains by the end of segmentation stages. A previous study in zebrafish concluded that *lbx2 expression is restricted to ventral regions of the somites *[23]. This discrepancy could be due to the rather weak expression of *lbx2 *in the epaxial domain (Fig. 1E), which may have been missed in the earlier study. By 48 hpf, expression of *lbx2 *disappears from somites in the trunk. Similarly in *Xenopus*, the ventrolateral region of rostral trunk somites, which contains precursors of hypaxial body wall, expresses *lbx1*, and this expression later becomes weak and eventually lost [15]. Satellite cells in mouse that express *Lbx1 *also extinguish expression during myogenic differentiation [34]. Thus, *Lbx *gene expression may be transient during myogenesis in all species.

During muscle development in chick, the myotome forms in three waves [35,36]. The first wave of early post-mitotic progenitors appears along the entire dorsomedial aspect of the epithelial somite. Then in the second wave, cells migrate from all four lips of the dermomyotome, although myofibers are generated from only the rostral and caudal edges. During the third wave, there is a balance between differentiation and proliferation. Similarly, zebrafish muscle development can be divided into several steps. First, epithelial adaxial cells, precursors of slow and muscle pioneers, are specified to be slow or muscle pioneers by Hh signaling from the notochord [19]. Then, slow muscle precursors migrate to the lateral surface of the somite [18]. Second, a wave of fast muscle morphogenesis is induced by the migration of the slow muscle precursors [37]. Finally, new slow-muscle fibers are added in growth zones near the dorsal and ventral extremes of the myotome, and this muscle growth continues into larval life [38]. We demonstrated that *lbx2 *expression first appears in adaxial cells. Adaxial cells lose *lbx2 *expression before they incorporate into the somite. Then, a subset of fast muscle precursors begins to express *lbx2*. Further analysis is required to determine whether *Lbx2 *is transiently expressed in all or only a subset of muscle precursors and whether such transient expression is linked to particular waves of muscle development.

### Migration of slow muscle precursors in Lbx2 knockdown embryos

In mouse, disruption of Lbx1 causes a severe loss of limb muscle, because muscle progenitors fail to migrate [13]. We showed that disruption of Lbx2 activity causes decreased *myod *expression in fin bud and hyoid muscles (Fig. 3), suggesting that Lbx2 could be involved in migration of these precursors in zebrafish. Additionally, we previously reported that a subset of adaxial cells migrate laterally to form slow muscle [18]. The expression of *lbx2 *in adaxial cells raised the possibility that Lbx2 may regulate migration of slow muscle precursors, too. Although transplantation analysis showed that the early movement of cells lacking Lbx2 is disrupted during gastrulation (see Additional file 1), slow muscle precursors eventually migrate radially (Fig. 4). Therefore, although it is likely that Lbx2 contributes to the migration of fin and head muscle precursors, Lbx2 is not required for migration of slow muscle precursors.

### A molecular link between Lbx2, a transcriptional repressor, and filament gene expression

The Lbx1 protein contains the engrailed repressor domain [39] and Lbx1 protein that lacks the engrailed domain fails to suppress *myod *expression in *Xenopus *[15]. Moreover, Lbx1 interacts with Corl1, a transcriptional corepressor [40]. These observations strongly suggest that Lbx1 functions as a transcriptional repressor. We find that Lbx2 that lacks the engrailed domain fails to induce *atca1 *and *mybpc1 *in *lbx2*-MO injected embryos, and also fails to suppress *mef2c *and *myogenin *in *lbx2*-MO injected embryos (Fig. 7). This result indicates that the engrailed repressor domain is required for function of Lbx2 in the formation of myofibrils. However, our observation that *lbx2*-MO leads to a decrease in filament gene expression suggests that filament genes cannot be direct targets of Lbx2. Instead, this result indicates that Lbx2 acts through an intermediate factor (or factors) to regulate expression of filament genes.

Myogenin and Mef2 are known to function in filament formation [7,41,42], and we showed that expression of both *myogenin *and *mef2c *is reduced by overexpression of Lbx2. Thus, Myogenin and Mef2c may act downstream of Lbx2 to regulate myofilament gene expression during formation of myofibrils. However, Myogenin and Mef2c are thought to function as transcriptional activators. Our observations that *myogenin *and *mef2c *expression is upregulated in *lbx2*-MO injected embryos, whereas filament gene expression is decreased by *lbx2*-MO, suggest that there must be other factors downstream of Lbx2, most likely additional transcriptional repressors. Thus, further studies are required to identify the proximal downstream mediators of Lbx2 function.

## Conclusion

Several steps are required for muscle progenitors to form functional skeletal muscle: (1) formation of a population of multipotent mesodermal cells, (2) specification and commitment to a muscle fate, and (3) differentiation accompanied by cell fusion and assembly of contractile myofibrils. The precise timing of these steps requires a fine balance between transcriptional activation and repression [43,44]. Although many transcriptional activators and repressors involved in commitment, specification, and differentiation of muscle precursors have been identified [45], the transcriptional network that regulates terminal differentiation is still incompletely understood. Our study has identified Lbx2 as a regulator of myofibril formation through its action on expression of myofilament genes, including *myosin *and *actin*. A recent study showed that undifferentiated satellite cells express Lbx1, and Lbx1 expression diminishes as cells differentiate [34]. In addition, it is known that Lbx1 upregulates Pax7 expression and downregulates Myod in satellite cells [34], and we also find suppression of *pax7 *expression in *lbx2*-MO injected embryos (see Additional file 6). Thus, it is possible that Lbx1 maintains the immature state of satellite cells by regulating Pax7. Based on these observations, we suggest that the general function of Lbx2 may be to aid differentiation of myoblasts by promoting fusion and myofilament gene expression.

## Methods

### Animals

Embryos were obtained from the University of Oregon zebrafish facility, produced using standard procedures [46] and staged according to standard criteria [47]. The wild-type line used was AB.

### Plasmid construction

Gene-specific primers to amplify full-length zebrafish *lbx2 *(AJ29516) and part of *lbx1b *cDNA were designed based on the available Ensemble zebrafish genome assembly (Zv4).

Full-length zebrafish *lbx2 *was obtained from 24 post-fertilization (hpf) embryos by RT-PCR with primers lbx2 F (5'-ATG ACC TCC AGC TCT AAA GA-3') and lbx2 R (5'-TTA ATC GTC GAC CTC GAT TT-3').

The PCR product was cloned into the *Eco*RI site of pCS2 (pCS2-*lbx2*) and pCS2+EnR (pCS-*EnR-lbx2*) [48]. To make removed engrailed domain, amino acids 47–257 of zebrafish *lbx2 *was amplified by PCR with the primers lbx2 Δ En (5'-GGT ACC ATG ATC TTA AAC AAG CCC TCC GTT) and lbx2 R, and cloned into the *Asp*718 and *Xba*I site of pCS (pCS-*lbx2 *Δ *En domain*). A part of zebrafish *lbx1b *was obtained from 24 post-fertilization (hpf) embryos by RT-PCR with primers lbx1bF (5'-CTCCACCTGCTAACTCAAAC3') and lbx1b R(5'-TCAGTCATCTACATCAATTTCCTCG-3'). Zebrafish genome informatics analysis (based on the Zv6 assembly) reveals that *lbx1b *lies chromosome 13 at location 20, 761, 204–20, 762, 898. basepairs (bp) and *lbx2 *lies on chromosome 14 at location 1, 185, 681–1, 188, 749 bp [22].

### *In vitro *mRNA synthesis

Capped mRNAs were transcribed from linearized DNA templates with SP6 RNA polymerase *in vitro *transcription kits (mMESSAGE mMACHINE SP6, Ambion, Inc., Austin, TX USA) according to the manufacturer's instructions. pCS2-*lbx2 *plasmid was linearized with *Xba*I. pCS2-*EnR-lbx2 *plasmid was linearized with *Asp*718. pCS-*lbx2 *Δ *En domain *was linearized with *Xba*I.

### *In situ *mRNA hybridization

The *in situ *labeling was performed as previously described [49] using the markers: *myod, myf5, myogenin *[27], *no tail (ntl) *[50], *skeletal muscle alpha-actin *(*acta1*)*, skeletal muscle troponin T *(*tnnt*: currently named *skeletal slow troponin T1, tnnt1), fast skeletal muscle tropomyosin *(*tpma*), *skeletal fast troponin T3b *(*tnntt3b*)*, fast skeletal muscle troponin C *(*tnnc*) [31,24] (ZFIN), *skeletal muscle myosin heavy chain *(currently named *myosin, skeletal muscle heavy polypeptide 1, myhz1*), *slow myosin heavy chain *1 (smyhc1)*, slow type myosin binding protein C (mybpc1), fast skeletal muscle myosin light chain 2 *(currently named *skeletal muscle myosin light polypeptide 2, mylz2*)*, fast muscle specific myosin heavy polypeptide 2 (myhz2) *[7,31,32]. Probes were synthesized using SP6 RNA polymerase or T7 RNA polymerase. Embryos processed for whole-mount in situ hybridization were photographed using a Leica MZFGIII microscope and Axiocam digital camera.

### Microinjection

mRNA was dissolved in double distilled H_2_O to final concentrations of 30 ng/μl to 50 ng/μl. Phenol red was added to the solution. Approximately 1 nl of RNA or DNA was injected at the one-cell stage using published procedures [51]. *lbx2*-MO was directed to the translation start site, splice donor site (Gene Tools, LLC)*, lbx2-ATG *MO: TCATGTCTTTAGAGCTGGAGGTCAT, *lbx2-splice donor *MO: TTATGAACTTTTTACCTTCTGCTGC, *lbx1b-splice donor *MO: ACACCGGGCCTTGTGTTTACCTTCT. Stock solutions were resuspended at 2.5 μg/μl.

To obtain mosaic expression, we performed the plasmid injected (100 ng/μl).

### Antibody labeling and quantification of muscle fiber numbers

Labeling with F59, EB165, 4D9 and Prox1 was as previously described [3,5,17]. The primary antibodies were mAb F59 (anti-MyHC, slow muscle) at 1:20, mAb EB165 (anti-MyHC, fast muscle) at 1:5000, mAb 4D9 (anti-Engrailed) at a dilution of 1:20, rabbit anti-Prox1 (AngioBio Co.) at a dilution of 1:500. Secondary antibodies were AlexaFluor-594 goat anti mouse IgG at 1:1000 and AlexaFluore-488 goat anti-rabbit IgG at 1:1000 (Molecular Probes). The images ware collected using a LSM 5 PASCAL confocal microscope configured around an AXIO Imager M1 upright microscope. The numbers of slow muscle and muscle pioneers were calculated from the total number of cells labeled by the Prox1 antibody (slow muscle) and the total number of cells labeled by both the Prox1 and 4D9 antibodies (muscle pioneer cell) per embryo counted in four somites over the extended yolk at 24 hpf. Data represent the average number of slow muscle cells or muscle pioneer cells per somite. To quantify the phenotypes of muscle fibers, we examined somites over the extended yolk in 3–5 embryos. Fibers that were positioned in the middle of the dorsal half of the somite were measured. The lengths of muscle fibers were measured from the anterior somite border to the end of each fiber (Fig. 4B arrow). Because fiber thickness depends on how many sarcomeres line up in parallel [52], we measured the diameter of each fiber from its dorsal to its ventral edge (Fig. 4B arrowhead).

## Authors' contributions

HO carried out all experiments and analyzed the data. HO and MW designed and directed the project and wrote the manuscript. All authors read and approved the final manuscript.

## Supplementary Material

Additional file 1 **Cells with impaired motility due to Lbx2 knockdown still express *myod*.***lbx2*-MO and rhodamine (red) injected cells were transplanted into the margin, 75 degrees ventral to the shield, at shield stage. When control cells are transplanted into control embryos, the cells become distributed along the rostral caudal axis (A) and transplanted cells adjacent to the notochord express *myod *(A inset, arrow, ctrl > ctrl: n = 4). In contrast, transplanted *lbx2*-MO injected cells stay clumped and do not distribute along the rostral caudal axis, although cells adjacent to the notochord express *myod *normally (B inset, arrow, *lbx2*-MO > ctrl: n = 4/4). This effect on migration appears to be cell-autonomous because control cells transplanted into *lbx1b*-MO injected embryos behave normally (C, ctrl > *lbx2*-MO: n = 2). *lbx2*-MO + *lbx2*-mRNA injected cells become distributed along the rostral caudal axis (D, n = 13/15 rescued). Arrows indicate *myod *expressing transplanted cells. N: notochord. (A-D) Whole-mount embryos, dorsal views, rostral toward the top. Scale bar: 200 μm. Click here for file

Additional file 2 **Absence of Lbx2 activity results in malformation of slow and fast muscle fibers in *lbx2 *translation MO injected embryos.** (A, B, E, F) Control embryos. (C, D, G, H) *lbx2 *translation MO injected embryos. Embryos labeled with the slow muscle marker, F59 (A-D) and the fast muscle marker, EB165 (E-H). (A-H) Whole mounts, lateral views, rostral toward the left, dorsal toward the top. Scale bars: (A, C, E, G) 50 μm, (B, D, F, H) 20 μm. Click here for file

Additional file 3 **Expressivity of loss and gain of function Lbx2 phenotypes assayed by measuring rostral-caudal extension or dorsoventral thickness of muscle fibers.** Data are included for 24 hpf or 48 hpf control embryos or embryos injected with *lbx2 *morpholino or *lbx2 *mRNA. Click here for file

Additional file 4 **Lbx2 regulates slow and fast muscle fiber formation.** (A-B) Embryos labeled with α-actin (red) and F59, slow MyHC (green). Dotted lines indicate somite borders. 24 hpf embryos. (C-H) Embryos labeled with the slow muscle marker, F59 (C, D, E) or the fast muscle marker EB165 (F, G, H). 48 hpf embryos. (A-H) Whole mounts, lateral views, rostral toward the left, dorsal toward the top Scale bar: (A, B) 12.5 μm; (C-H) 50 μm. Click here for file

Additional file 5 **Expressivity of loss and gain of function Lbx2 phenotypes monitored by counting fast muscle nuclei.** Data are included for 24 hpf or 48 hpf control embryos or embryos injected with *lbx2 *morpholino or *lbx2 *mRNA. ^a^For the three groups, the number of nuclei was not significantly different (Kruskal-Wallis test, p = 0.1509). ^b^The number of nuclei at 48 hpf was significantly different (Kruskal-Wallis test, p = 0.0121) among the three groups. Further tests showed that the number of nuclei in *lbx2*-MO injected embryos at 48 hpf is significantly less than the others (Dunn's multiple comparison: ctrl = *lbx2 *mRNA: 0.280: p > 0.5, ctrl > *lbx *MO: 2.629: 0.05 > p > 0.02, *lbx *mRNA > *lbx *MO: 2.562: 0.05 > p > 0.02). Click here for file

Additional file 6 **myogenin, mef2ca and pax7 are downstream targets of Lbx2.** (A-C) Expression of *myogenin *(A), *mef2ca *(B) and *pax7 *(C) in control (ctrl), *lbx2*-MO injected embryos and *lbx2 *mRNA injected embryos. The expression of *myogenin *(A) and *mef2ca *(B) are suppressed by overexpression of *lbx2 *mRNA. In contrast, expression of *pax7 *is suppressed by *lbx2*-MO (C). *myogenin *(A, ctrl: 14/14 with normal expression, *lbx2*-MO: 13/15, *lbx2*-mRNA: 0/18), *mef2ca *(B, ctrl: 11/11, *lbx2*-MO: 4/5, *lbx2*-mRNA: 0/8), pax7 (D, ctrl: 7/7, lbx2-MO: 0/14, lbx2-mRNA: 13/13). (A-F) Whole mounts, lateral views, rostral toward the left, dorsal toward the top. Scale bar: 100 μm. Click here for file
