# Supplementary figures and images for: Lbx2 regulates formation of myofibrils
Source: BMC Dev Biol. 2009 Feb 12;9:13. doi: 10.1186/1471-213X-9-13 (PMC2656488; doi:10.1186/1471-213X-9-13)

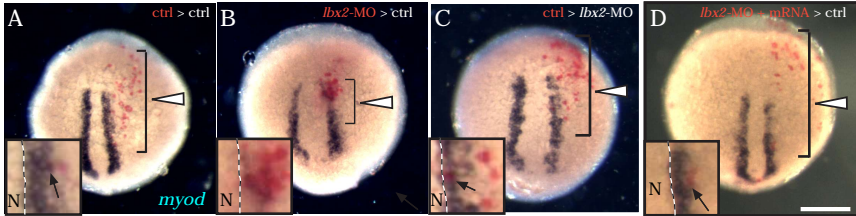

Additional file 1

Supplement: Additional file 1 — Cells with impaired motility due to Lbx2 knockdown still express myod.lbx2-MO and rhodamine (red) injected cells were transplanted into the margin, 75 degrees ventral to the shield, at shield stage. When control cells are transplanted into control embryos, the cells become distributed along the rostral caudal axis (A) and transplanted cells adjacent to the notochord express myod (A inset, arrow, ctrl > ctrl: n = 4). In contrast, transplanted lbx2-MO injected cells stay clumped and do not distribute along the rostral caudal axis, although cells adjacent to the notochord express myod normally (B inset, arrow, lbx2-MO > ctrl: n = 4/4). This effect on migration appears to be cell-autonomous because control cells transplanted into lbx1b-MO injected embryos behave normally (C, ctrl > lbx2-MO: n = 2). lbx2-MO + lbx2-mRNA injected cells become distributed along the rostral caudal axis (D, n = 13/15 rescued). Arrows indicate myod expressing transplanted cells. N: notochord. (A-D) Whole-mount embryos, dorsal views, rostral toward the top. Scale bar: 200 μm. [file 1471-213X-9-13-S1.pdf]

Slow

Slow

Fast

Fast

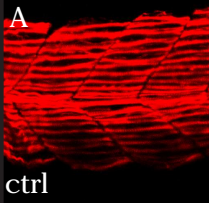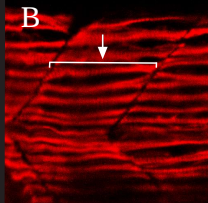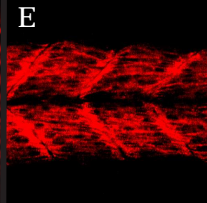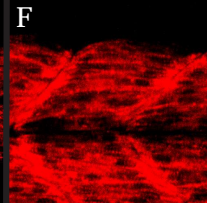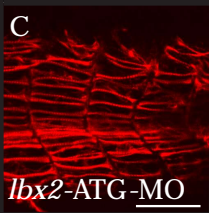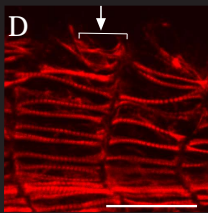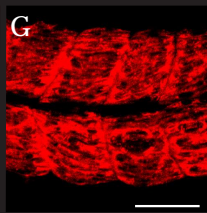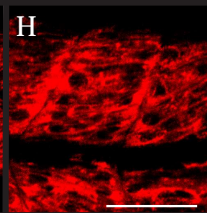

Additional file 2

Supplement: Additional file 2 — Absence of Lbx2 activity results in malformation of slow and fast muscle fibers in lbx2 translation MO injected embryos. (A, B, E, F) Control embryos. (C, D, G, H) lbx2 translation MO injected embryos. Embryos labeled with the slow muscle marker, F59 (A-D) and the fast muscle marker, EB165 (E-H). (A-H) Whole mounts, lateral views, rostral toward the left, dorsal toward the top. Scale bars: (A, C, E, G) 50 μm, (B, D, F, H) 20 μm. [file 1471-213X-9-13-S2.pdf]

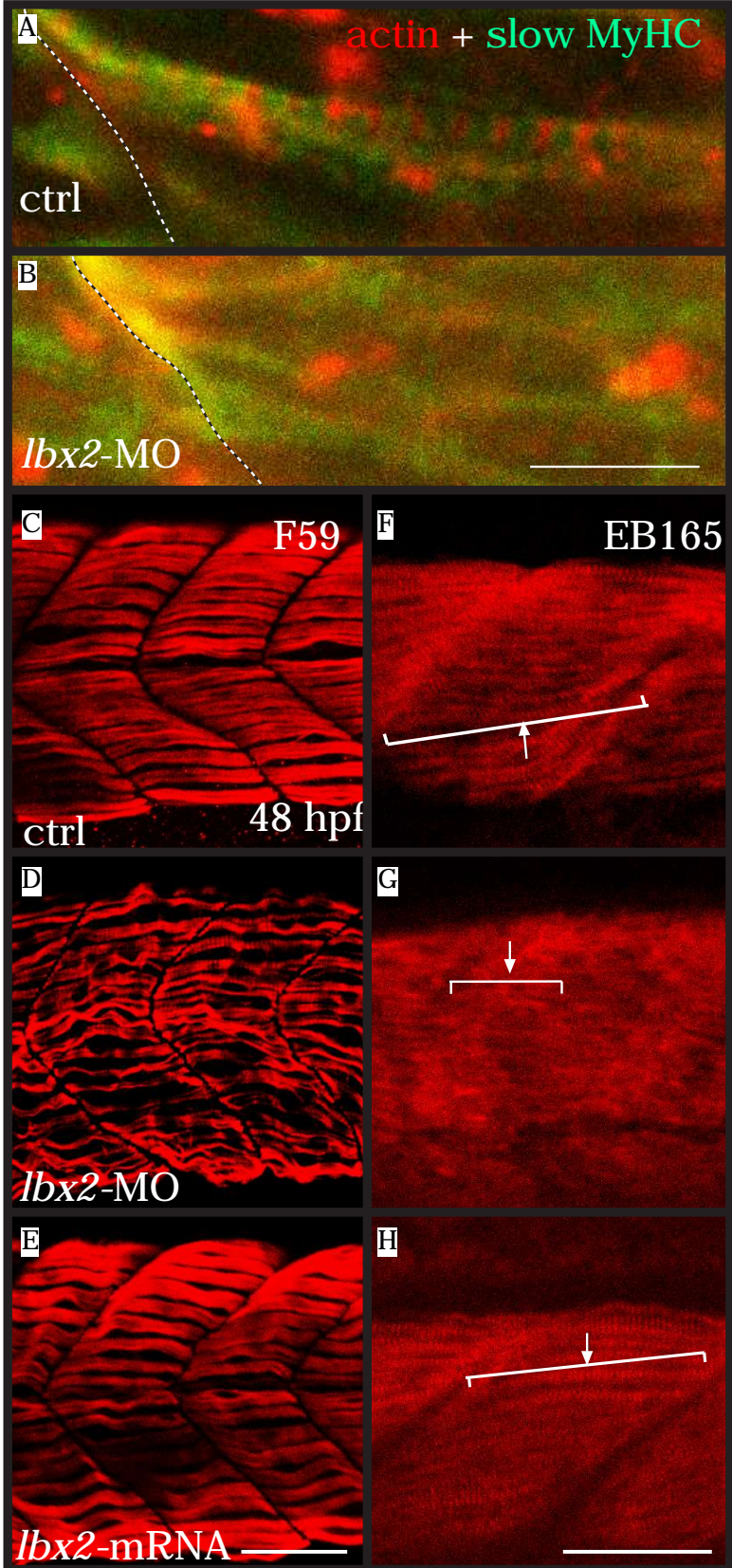

Supplement: Additional file 4 — Lbx2 regulates slow and fast muscle fiber formation. (A-B) Embryos labeled with α-actin (red) and F59, slow MyHC (green). Dotted lines indicate somite borders. 24 hpf embryos. (C-H) Embryos labeled with the slow muscle marker, F59 (C, D, E) or the fast muscle marker EB165 (F, G, H). 48 hpf embryos. (A-H) Whole mounts, lateral views, rostral toward the left, dorsal toward the top Scale bar: (A, B) 12.5 μm; (C-H) 50 μm. [file 1471-213X-9-13-S4.pdf]

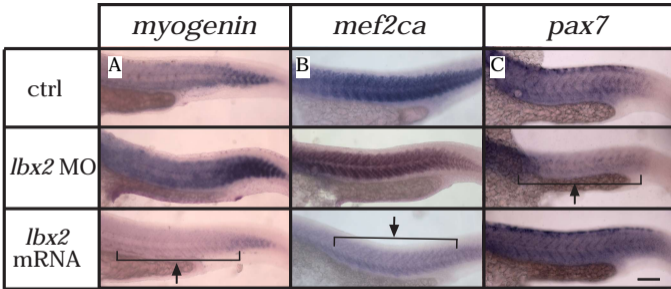

Additional file 6

Supplement: Additional file 6 — myogenin, mef2ca and pax7 are downstream targets of Lbx2. (A-C) Expression of myogenin (A), mef2ca (B) and pax7 (C) in control (ctrl), lbx2-MO injected embryos and lbx2 mRNA injected embryos. The expression of myogenin (A) and mef2ca (B) are suppressed by overexpression of lbx2 mRNA. In contrast, expression of pax7 is suppressed by lbx2-MO (C). myogenin (A, ctrl: 14/14 with normal expression, lbx2-MO: 13/15, lbx2-mRNA: 0/18), mef2ca (B, ctrl: 11/11, lbx2-MO: 4/5, lbx2-mRNA: 0/8), pax7 (D, ctrl: 7/7, lbx2-MO: 0/14, lbx2-mRNA: 13/13). (A-F) Whole mounts, lateral views, rostral toward the left, dorsal toward the top. Scale bar: 100 μm. [file 1471-213X-9-13-S6.pdf]
