# Supplementary material for: Lbx2 regulates formation of myofibrils
Source: BMC Dev Biol. 2009 Feb 12;9:13. doi: 10.1186/1471-213X-9-13 (PMC2656488; doi:10.1186/1471-213X-9-13)
Supplement: Additional file 3 — Expressivity of loss and gain of function Lbx2 phenotypes assayed by measuring rostral-caudal extension or dorsoventral thickness of muscle fibers. Data are included for 24 hpf or 48 hpf control embryos or embryos injected with lbx2 morpholino or lbx2 mRNA. [file 1471-213X-9-13-S3.pdf]

Stage		Rostral-caudal length of slow muscle fiber (μm)											Average of length	Total number of counted fiber
24 hpf	ctrl	58.86	59.35	56.74	61.00	55.69	64.00	62.06	57.14				59.65	8
	lbx-MO	55.07	52.10	35.10	30.04	20.76	29.99	15.96	26.65	24.75			32.27	9
	lbx mRNA	58.58	57.75	59.61	59.95	46.52	45.70	48.70	48.93				53.09	8
48 hpf	ctrl	55.73	57.37	60.51	67.29	60.73	74.18	50.20	47.51	49.82			58.15	9
	lbx-MO	42.92	34.21	42.42	38.63	49.29	49.15	52.54	48.42	53.56	57.28		45.68	10
	lbx mRNA	51.63	65.61	55.41	65.45	54.29	61.32	49.29	48.54	51.89			55.93	9
		Dorsoventral thickness of slow muscle fiber												
24 hpf	ctrl	5.34	2.47	5.36	3.39	3.49	3.19	3.49	2.47	2.29	2.58	2.67	1.21	11
	lbx-MO	1.34	1.13	1.64	1.34	1.15	1.95	1.64	1.64	2.05	1.54		1.53	10
	lbx mRNA	2.36	3.60	2.57	3.70	2.26	2.67	3.39	2.88				2.92	8
48 hpf	ctrl	6.90	4.44	5.60	4.77	4.73	4.13	4.27	5.20	5.92			5.11	9
	lbx-MO	3.29	1.97	4.60	2.30	5.73	2.14	3.12	3.79	2.30	3.46		3.46	9
	lbx mRNA	5.26	4.61	5.42	5.59	6.41	5.92	5.59	5.59	4.77			5.46	8
		Rostral-caudal length of fast muscle fiber												
24 hpf	ctrl	48.10	56.25	53.85	51.73	56.40	50.12	55.70					52.98	7
	lbx-MO	17.79	38.81	45.21	29.07	34.08	31.80	20.64	18.67				29.52	8
	lbx mRNA	61.40	59.44	68.45	63.11	57.32	56.87	67.47					61.07	7
48 hpf	ctrl	79.54	75.34	66.50	70.73	83.15	109.79	92.27	103.74				85.14	8
	lbx-MO	56.55	68.08	59.55	65.42	68.22	60.29	60.51					60.51	7
	lbx mRNA	76.19	84.68	85.23	86.93	85.37	100.25	80.80	90.91				86.30	8
		Dorsoventral thickness of fast muscle fiber												
24 hpf	ctrl	2.56	1.28	1.56	1.74	2.28	1.11	2.38	1.83				1.84	8
	lbx-MO	2.37	1.06	1.34	1.66	2.77	1.90	1.19	1.98				1.78	8
	lbx mRNA	0.77	1.03	0.88	1.37	1.20	0.86	1.13	1.80				1.13	8
48 hpf	ctrl	2.60	2.47	2.61	1.92	1.92	2.47	2.20	3.02				2.40	8
	lbx-MO	2.19	2.47	2.33	1.92	3.29	3.63	3.83	2.12				2.72	8
	lbx mRNA	2.47	3.68	3.12	2.69	3.29	3.29	3.14	3.78				3.18	8

Additional file 3
