# Supplementary material for: Lbx2 regulates formation of myofibrils
Source: BMC Dev Biol. 2009 Feb 12;9:13. doi: 10.1186/1471-213X-9-13 (PMC2656488; doi:10.1186/1471-213X-9-13)
Supplement: Additional file 5 — Expressivity of loss and gain of function Lbx2 phenotypes monitored by counting fast muscle nuclei. Data are included for 24 hpf or 48 hpf control embryos or embryos injected with lbx2 morpholino or lbx2 mRNA. aFor the three groups, the number of nuclei was not significantly different (Kruskal-Wallis test, p = 0.1509). bThe number of nuclei at 48 hpf was significantly different (Kruskal-Wallis test, p = 0.0121) among the three groups. Further tests showed that the number of nuclei in lbx2-MO injected embryos at 48 hpf is significantly less than the others (Dunn's multiple comparison: ctrl = lbx2 mRNA: 0.280: p > 0.5, ctrl > lbx MO: 2.629: 0.05 > p > 0.02, lbx mRNA > lbx MO: 2.562: 0.05 > p > 0.02). [file 1471-213X-9-13-S5.pdf]

Stage		Number of cells			Average of nuclei per cell	Total number of counted cell	Number of embryos
		Number of nuclei					
		1	2	3			
24 hpf	ctrl[a]	1	12	6	3.21	19	4
	lbx-MO[a]	9	5	5	2.43	14	4
	lbx mRNA[a]	4	10	6	2.1	20	4
48 hpf	ctrl[b]	1	9	10	2.45	20	4
	lbx-MO[b]	7	10	4	1.85	21	4
	lbx mRNA[b]	2	13	10	2.35	25	4

Additional file 5
